# Bilateral Vision Loss after Delivery in Two Cases: Severe Preeclampsia and HELLP Syndrome

**DOI:** 10.4274/tjo.45722

**Published:** 2015-12-05

**Authors:** Gökhan Çelik, Ahmet Eser, Murat Günay, Nursal Melda Yenerel

**Affiliations:** 1 Zeynep Kamil Women and Children’s Diseases Education and Research Hospital, Clinic of Ophthalmology, İstanbul, Turkey; 2 Zeynep Kamil Women and Children’s Diseases Education and Research Hospital, Clinic of Obstetrics and Gynecology, İstanbul, Turkey; 3 Haydarpaşa Numune Education and Research Hospital, Clinic of Ophthalmology, İstanbul, Turkey

**Keywords:** Macular hemorrhage, serous retinal detachment, HELLP syndrome, preeclampsia

## Abstract

Two patients with no symptoms of hypertension in their medical history before pregnancy were referred to the obstetrics emergency clinic with hypertension and visual complaints. After physical examination and laboratory tests, one of the patients was diagnosed with severe preeclampsia while the other was diagnosed with HELLP syndrome (Hemolysis-Elevated Liver enzymes-Low Platelets). Ocular examinations were performed after delivery due to the patients’ worsening visual complaints. The severe preeclamptic patient showed bilateral serous retinal detachment (SRD) while the patient with HELLP syndrome showed bilateral macular hemorrhage. Systemic blood pressure control was advised. The patients’ ocular findings and visual acuities improved in the follow-up periods. SRD and macular hemorrhage can be observed in patients with preeclampsia and HELLP syndrome as a result of the disruption of retinal and choroidal vasculature.

## INTRODUCTION

Preeclampsia, the most common medical complication of pregnancy, is characterized by elevated systemic blood pressure (≥140/100 mmHg), proteinuria (≥300 mg/24 hours) and generalized edema, and typically appears after the 20th week of pregnancy. Its occurs in 5-10% of all pregnancies.^1^ Severe preeclampsia presents with systemic blood pressure over 160/110 mmHg, proteinuria ≥2 gr/24 hours, serum creatinine >2 mg/dl, oliguria, thrombocytopenia, epigastric pain, cerebral and visual disruptions, headache, pulmonary edema, and elevated liver enzymes. Convulsions before or after birth in addition to the symptoms of preeclampsia indicates progression to eclampsia. HELLP (Hemolysis-Elevated Liver enzymes-Low Platelets) syndrome, which is a more severe form that develops in approximately 4-20% of preeclamptic patients, is characterized by hemolysis, elevated liver enzymes and low thrombocyte numbers, and is associated with high maternal and perinatal morbidity and mortality. HELLP syndrome was first mentioned in 1954 by Prichard and was described clinically in detail in 1982 by Weinstein.^2^ It generally occurs in the third trimester, but more rarely can develop in earlier weeks of pregnancy or in the postpartum period, and may appear without hypertension.

Preeclampsia or HELLP syndrome patients may develop retinal and choroidal circulation dysfunction, and various fundoscopic findings and subsequent vision loss may occur as a result. These patients may have severe hypertensive retinopathy findings such as retinal hemorrhage, subretinal serous fluid accumulation, papilledema, and Elschnig spots.^3^

In this study we present the concurrent fundus findings of one case of severe preeclampsia and one case of HELLP syndrome.

## CASE REPORTS

### Case 1

**Figure 1:**
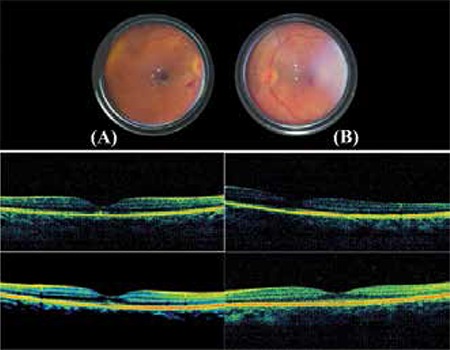
Case 1, fundus images (taken with mobile phone) of the patient diagnosed with HELLP on postpartum day 2 in the intensive care unit. Macular hemorrhage, retinal hemorrhage and cotton wool spots in the left eye (A) and macular hemorrhage and cotton wool spots in the right eye (B) are visible. Optical coherence tomography at 1 month and 1 year were normal

A 37-year-old patient in the 32nd week of her second pregnancy presented to our hospital with headache and blurred vision. At initial examination, systemic blood pressure was 220/110 mmHg and bilateral (++++) pretibial edema was observed during physical examination. Laboratory tests revealed thrombocytopenia (72x103/mm3) and liver function tests were elevated: alanine aminotransferase (ALT), 104 IU/L; aspartate aminotransferase (AST), 129 IU/L; and lactate dehydrogenase (LDH), 603 U/L. Urine analysis showed (+++) proteinuria. On obstetric ultrasonography the fetus showed symmetrical developmental delay consistent with 29 weeks. The patient was diagnosed with HELLP syndrome and an emergency cesarean section was performed. On ophthalmologic examination conducted in the intensive care unit on postpartum day 2, the patient’s visual acuity was counting fingers at 5 meters. Bilateral light reflexes were natural, anterior segment examination revealed no irregularities in either eye, and intraocular pressure was within normal range. Retinal hemorrhage and bilateral macular hemorrhage with cotton wool spots were observed during fundus examination. Systemic blood pressure regulation and follow-up were recommended. In postpartum week 1 the patient’s best corrected visual acuity (BCVA) was 2/10 in the right and 5/10 in the left eye. At 1-month follow-up examination the hemorrhages had completely resolved, BCVA was 8/10 in the right and 10/10 in the left eye, and optical coherence tomography (OCT) was normal. At 1-year follow-up, both eyes had perfect visual acuity, examination revealed no pathology, and OCT was normal (Figure 1).

### Case 2

**Figure 2:**
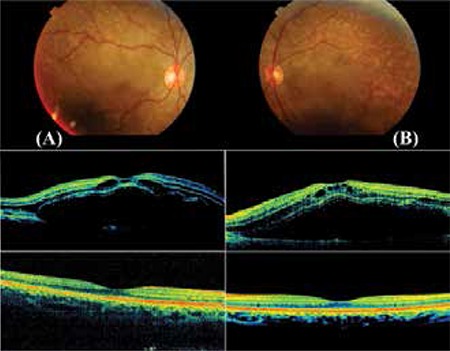
Case 2, postpartum day 5 colored fundus photograph from severe preeclamptic patient showing serous retinal detachment accompanied by many yellow, opaque lesions in the retinal pigment epithelium. Elevation of the inner and outer retinal layers associated with severe neurosensorial retinal detachment and intraretinal cysts were noted in optical coherence tomography images taken at the same time. Complete resolution of the macular edema and normal foveal contours were observed on follow-up optical coherence tomography at 1 year

A 20-year-old patient in her first, unfollowed pregnancy presented to our hospital with headache and blurred vision. Initial systemic blood pressure was 180/100 mmHg and bilateral (++) pretibial edema was observed on physical examination. Obstetric ultrasonography showed the fetus was consistent with 39 weeks gestation. Thrombocyte count was 176x103/mm3, ALT was 11 IU/L, AST was 28 IU/L, and urine analysis showed (+++) proteinuria. The patient was admitted to the hospital with a diagnosis of severe preeclampsia and cesarean section was performed. The patient had a BCVA of 2/10 in both eyes on ophthalmologic examination on postpartum day 1. Anterior segment examination findings were normal in both eyes and intraocular pressure was within normal range. Fundus examination revealed bilateral macular serous retinal detachment (SRD) and areas of serous elevation in the peripheral retina. On postpartum day 3, the patient’s vision had decreased to counting fingers at 1 meter and fundus examination revealed widespread SRD at the macula and peripheral retina. OCT done at the same time showed elevation of the inner and outer retinal layers associated with severe neurosensorial retinal detachment as well as intraretinal cysts. Due to these findings, systemic blood pressure control and follow-up were advised. At 1-month follow-up the patient’s vision had fully recovered. At final 1-year follow-up, vision was normal and no irregularities were observed during examination. OCT showed complete regression of the macular SRD and normal foveal contours (Figure 2).

## DISCUSSION

SRD occurs in approximately 1% of preeclampsia and HELLP syndrome cases. SRD may be unilateral or bilateral and may appear before birth or in the postpartum period.^4,5^ Other findings such as central retinal vein occlussion, vitreous hemorrhage and cortical blindness have also been reported in HELLP syndrome.^6,7,8^ Different theories are proposed for the mechanism of SRD development in disease. One theory is that SRD results from choroidal ischemia; another is that it develops due to subretinal fluid accumulation secondary to increased vascular permeability. SRD in preeclampsia is attributed to damage to choroidal vessels and small-to-medium diameter vessels in the choriocapillaris resulting from severe hypertension.^9,10^ Another study from Turkey reported fundus fluorescein angiography findings consistent with choroidal ischemia in a preeclamptic patient with bilateral SRD.^11^ Lin et al.^12^ performed wide-field angiography in a preeclamptic patient with HELLP syndrome and bilateral serous macular detachment and found peripheral retinal leakage, which they attributed to choroidal ischemia as well as blood-retina barrier dysfunction. In our preeclamptic patient, we also observed bilateral serous macular detachment and retinal SRD, especially in the inferior region. Furthermore, widespread ischemic lesions consistent with hypertensive retinopathy at the choroid level were observed on color fundus photographs. However, the patient’s fundus findings completely resolved within 1 month. Similarly, Atış et al.^13^ reported bilateral SRD in a patient with severe preeclampsia, and the patient’s vision completely recovered after 6 weeks.

In the literature, various findings have been identified in the ocular involvement of HELLP syndrome, especially the development of retinal hemorrhage due to lowered thrombocyte count has been reported. In a Turkish study, a case of bilateral concommitant macular hemorrhage was presented.^14^ One of our cases also had bilateral macular hemorrhage accompanied by areas of retinal hemorrhage and hypertensive retinopathy findings. A study of ocular findings in HELLP syndrome patients reported hypertensive changes in 16%, SRD in 3.7% and cortical blindness in 2.7% of cases.^15^ As stated in the literature, we also believe the retinal hemorrhage in our patient was associated with vascular changes arising in the disease due to other causes. In addition to the vasospasm commonly seen with HELLP, endothelial dysfunction, inflammation and altered chorioretinal circulation result in ischemia, which plays a role in the development of disease signs.^9^

In conclusion, various ocular findings may occur in preeclampsia and HELLP syndrome; in most cases, these findings spontaneously resolve with proper management of the disease. Visual symptoms in these patients should definitely be regarded as serious, and the patients should be referred to an ophthalmologist in a timely manner.
